# Exploring metaphor's communicative effects in reasoning on vaccination

**DOI:** 10.3389/fpsyg.2022.1027733

**Published:** 2022-11-18

**Authors:** Francesca Ervas, Pietro Salis, Cristina Sechi, Rachele Fanari

**Affiliations:** Department of Education, Psychology, Philosophy, University of Cagliari, Cagliari, Italy

**Keywords:** uncertain reasoning, metaphor, collective immunity, trust, vaccine communication, defeasible reasoning, vaccination

## Abstract

**Introduction:**

The paper investigates the impact of the use of metaphors in reasoning tasks concerning vaccination, especially for defeasible reasoning cases. We assumed that both metaphor and defeasible reasoning can be relevant to let people understand vaccination as an important collective health phenomenon, by anticipating possible defeating conditions.

**Methods:**

We hypothesized that extended metaphor could improve both the argumentative and the communicative effects of the message. We designed an empirical study to test our main hypotheses: participants (*N* = 196, 78% females; Meanage = 27.97 years, SDage = 10.40) were presented with a text about vaccination, described in either literal or metaphorical terms, based on uncertain vs. safe reasoning scenarios.

**Results:**

The results of the study confirmed that defeasible reasoning is relevant for the communicative impact of a text and that an extended metaphor enhances the overall communicative effects of the message, in terms of understandability, persuasion, perceived safety, and feeling of control over the health situation, collective trust in expertise and uptake of experts' advice. However, the results show that this effect is significantly nuanced by the type of defeasible reasoning, especially in the case of participants' trust in expertise and commitment to experts' advice.

**Conclusion:**

Both communicative and defeasible reasoning competences are needed to enhance trust in immunization, with possible different outcomes at an individual and collective level.

## Introduction

In times of the COVID-19 pandemic, the promotion of collective vaccination in institutional campaigns is of utmost importance. It is crucial for public health to make the vital need for collective vaccination as much clear as possible especially to hesitant people, as COVID-19 vaccination is on a voluntary basis in most countries. Vaccine hesitancy has been an important issue in institutional communication, especially in the context of social resistance to vaccination coverage and the diffusion of anti-vaccine movements. Even though some scholars argued that institutional communication might be irrelevant to nudge the population toward vaccination (MacDonald, 2015), inadequate institutional communication might contribute to spreading an irrational attitude toward vaccination and the refusal of their treatment against COVID-19. Indeed, Biasio et al. (2016, p. 2986) remarked that “communication based on valid and shared strategies, as well as on coherent behaviors, can modify the attitudes toward vaccinations, becoming one of the main components of the global strategy to oppose vaccine hesitancy”.

Citizens' understanding of how vaccination works should be considered fundamental in institutional communication, especially during pandemic times. Metaphors might be useful pedagogical devices in vaccine communication to explain a health phenomenon, which otherwise might remain unintelligible to laypeople. Metaphors can indeed be a way to grasp an unknown/less known concept (the target) by using a better-known concept (the source) (Lakoff and Johnson, 1980; Kövecses, 2002; Bowdle and Gentner, 2005; Gibbs, 2008). In particular, we consider metaphor as a reasoning device guiding the readers along a path of inferences to a conclusion, which attributes to the target some relevant properties of the source (Oswald and Rihs, 2014; Ervas, 2019). Metaphors in health communication have been widely used, especially in the case of cancer, but they have been notoriously criticized as violent uses of language for people suffering from cancer treatments (Semino et al., 2018). Scholars have proposed many metaphors to let people understand vaccination, ranging from the conventional military metaphor of the “garrison” to the novel metaphor of the “beehive”. The concept of “herd immunity” is also metaphorical, but people criticized its communicative entailments, pointing out that it made them feel like mindless sheep following the flock (Biss, 2014; Ervas, 2018). Each metaphor provides a specific perspective to interpret both the disease and its development, as it entails a framing effect on the health phenomenon to be explained (Semino et al., 2016, 2018). However, it is not clear whether and which metaphors better communicate how vaccination works and, at the same time, are helpful to achieving compliance in the management of COVID-19. Indeed, in the case of vaccination, it might be the case that, even though metaphors are effective devices for citizens' education, literal communication on vaccines and vaccine-preventable diseases is still preferable.

Exposure to metaphorical framing proved capable to modify how people reason about a specific social problem (Thibodeau and Boroditsky, 2011, 2013, but see Steen et al., 2014 for criticism), also in the case of metaphors used in vaccine communication (Scherer et al., 2015). In metaphor framing studies, participants are usually presented with a metaphorical (vs. non-metaphorical) description of a target issue and then asked to make a judgment or a decision on the target to check whether it is influenced by the metaphorical frame provided by the text. Thibodeau and Boroditsky (2011, p. 1) stated that “even the subtlest instantiation of a metaphor (*via* a single word) can have a powerful influence over how people attempt to solve social problems”, such as crime, even when presented with possible alternatives after reading the text (Thibodeau and Boroditsky, 2013). But later on, Steen et al. (2014) addressed some criticisms of their studies, which actually presented to participants extended metaphors, i.e., metaphors reinforced by other metaphorical words in the text, without a control (literal or “neutral framed”) condition. Steen et al. (2014), therefore, proposed a series of follow-up studies, also presenting texts with no additional support for the metaphoric frame, adding a non-metaphorical control condition, and previously measuring the political preferences of the participants. Based on previous studies (Thibodeau and Boroditsky, 2011, 2013), the authors checked indeed whether the metaphoric frame could influence the beliefs of the participants and consequently their decisions on the target, i.e. crime, either changing or reinforcing their political views, when consistent with the metaphoric frame. They found neither effects of the metaphorical frame nor of the metaphorical support on reasoning. They concluded that increased media attention and/or simple text exposure to the issue as a relevant social problem finally influenced policy preferences. Interestingly, they also found that the (conventional) metaphors like “beast” and “virus” did not “surpass a non-metaphorical frame in terms of prominence or attention” (Steen et al., 2014, p. 21), while the metaphorical support increases the activation, and thus the prominence of the metaphorical frame. They finally suggested that the novelty, artfulness, or deliberateness of a metaphor might play a major role in enhancing the metaphorical processing as well as the communicative effects of the text.

While Thibodeau and Boroditsky (2011) focused on conventional metaphors, Scherer et al. (2015) also presented texts with novel metaphors for their study on the metaphorical framing effect of flu description on vaccination intentions. They anyway found no significant difference in the metaphorical framing of vaccination intentions, “with novel and conventional metaphors all increasing vaccination intentions” (Scherer et al., 2015, p. 44). Though the metaphors were extended along the text *via* a relevant metaphorical property, the texts presented to participants were focused on the description of the flu in a reasoning situation that was consistent with the metaphorical frame. In other words, participants' disposition to get vaccinated was assessed *via* the reading of a text that did not directly bring participants to question the metaphorical frame and/or the relevant metaphorical property. As the results of the study showed, such reasoning scenarios do not change the (either against or in favor of vaccination) beliefs vaccination participants already strongly held, while having some impact on people who occasionally got the vaccination. However, in everyday life specific reasoning situations are widespread where laypeople question the (either metaphorical or literal) description of vaccination, possibly leading to undesired conclusions on vaccination as a collaborative endeavor. Some specific reasoning scenarios may indeed defeat conclusions about the necessity for vaccination to be collective, presenting further premises ranging from the phenomenon of single “free riders” to entire anti-vaccination communities. Such specific reasoning situations where vaccination is metaphorically described as collaborative have not been tested and are indeed relevant to understand whether and how metaphor interpretation interacts with the argumentative scaffolding of pro-vaccination texts. Certain reasoning tasks, concerning sets of premises whose consistency is not fully explicit, usually require a major effort to evaluate the conclusions. These cases can be called uncertain scenarios. This type of reasoning is usually framed in terms of defeasible reasoning, and these premises are usually defined as *defeaters*, as they defeat the conclusion of the argument (Pollock, 1970, 1974; Kelly, 2014). Especially when intentionally used, metaphors are supposed to be highly relevant in a variety of argumentative discourse structures (Van Poppel, 2020a,b), but they have not yet been tested for their communicative power in defeasible reasoning situations, as required by a full understanding of vaccination as a collaborative phenomenon.

Reasoning about vaccination can thus be fruitfully approached by trying to highlight the role that certain premises play in it. In fact, depending on certain premises, a pattern of inference can be more or less exposed to uncertainty. Deductive reasoning dictates that conclusions necessarily follow from its premises, while reasoning where the insertion of new premises defeats its conclusion is called *defeasible*. Ordinary reasoning is usually defeasible, but within a certain set of premises, there are premises that are in principle more likely to put the conclusion into question, or even defeat it (Pollock, 1970, 1974; Kelly, 2014). Certain sets of premises can indeed be more or less consistent, and when slight (or even serious, depending on the context) inconsistencies show up, the reasoning can be exposed to uncertainty and the conclusion can be (temporarily) retracted. These premises may become, in these situations, potentially invalidating conditions for the reasoning process, possibly leading to a retraction of the conclusion. For example, consider a generic set of premises, such as the fact X; the fact Y; and its conclusion: the foreseen outcome O. This set of premises (X,Y) can be seen as the unproblematic (or “safe”) case, where the expected outcome (O) is likely to happen. If we add a new premise, call it “the fact Z”, which can alter the consistency of the set of premises, the reasoning task would get complicated, even requiring tentative solutions. These complications in the reasoning task require some means to explore the role of certain premises. The metaphor here is a potentially useful reasoning device to interpret the new premises, facilitate and implicitly *check* the consistency of the premises within the set, and eventually *accomplish* the reasoning task: either by defeating the former conclusion or by temporarily retracting it, while waiting for further clues. However, no empirical study on the effects of metaphor concerning reasoning about vaccination has been conducted. The study presented in the paper aims to fill this gap, by investigating whether a metaphor intentionally used to provide the readers with a perspective on vaccination as a collaborative health phenomenon, might enhance the communicative effects of a text-based on reasoning about collective vaccination.

## Theoretical background

In this section, we will discuss two research assumptions. The first assumption is that ordinary reasoning, and therefore also reasoning about vaccination, is exposed to uncertainty (Salis and Ervas, 2021). The second assumption is that metaphor is a reasoning device to better understand the target, i.e., vaccination as a collective health phenomenon (Ervas, 2018).

### Uncertainty and reasoning in vaccine communication

Vaccine hesitancy and vaccine communication present a number of challenges to health experts' competence. Some of these challenges comprise reasoning about vaccination as a collective endeavor. In uncertain reasoning scenarios, some relevant premises have the potential logical role of defeaters, even if they do not actually defeat any conclusion on the necessity of vaccination as a collective effort. We, therefore, treat these cases as uncertain scenarios where defeats, if any, are yet to be established. The reasoning is uncertain when the presence or absence of a certain premise (or set of premises) is in general liable to alter, or even to put into question, its conclusion. This means that its conclusion does not necessarily follow from certain premises: if the premise P usually allows concluding C, a further premise F may defeat that reasoning; so, while the inference from P to C is valid in relatively easy scenarios, the eventual presence of F may defeat that transition, and C does not follow anymore, or at least it requires more effort on the side of the reasoner for proper assessment.

The fact that a premise may defeat reasoning, or make it uncertain, can be a problem as there is no warrant that this fact is known in advance. An example is the following: “collective vaccination is in place and effective in assuring that an unvaccinated child stays safe; Dave is an unvaccinated child; hence, Dave can stay safe”. This is a relatively certain scenario and quite an easy-going reasoning task. However, if we add certain premises, this conclusion may become uncertain, and can even be temporarily retracted. For example, we can add these premises: “Dave lives in a community with a high rate of vaccine hesitancy; an unvaccinated person cannot stay safe in a context of vaccine hesitancy”. These additional premises reshape the reasoning, to the point of defeating its conclusion. This reasoning would now involve a different series of steps: “collective vaccination is effective in assuring that an unvaccinated child stays safe; Dave is an unvaccinated child; Dave lives in a community with a high rate of vaccine hesitancy; an unvaccinated person cannot stay safe in a context of vaccine hesitancy”; hence, the conclusion “Dave can stay safe” does not follow anymore. The additional premises, that Dave lives in a community with a high rate of vaccine hesitancy and so forth, alter the set of premises and defeat the conclusion that we formerly were entitled to draw (Pollock, 1970, 1974). As this example shows, certain premises can systematically alter a much easier reasoning scenario making it uncertain and liable to be defeated. These potential complications can indeed lead to the temporary retraction of a conclusion, or to its global defeat.

We can distinguish two main roles that premises may play in uncertain scenarios: (a) premises that are liable to undercut the inference to a conclusion (U), as they may provide reasons that question the validity of a conditional, and (b) premises that are liable to rebut the conclusion (R), as they may provide reasons that directly question or even falsify a conclusion (as they can provide direct evidence of the falsity of the conclusion under scrutiny):

(a) The first role concerns the possibility of questioning the validity of a general relation between certain premises and a particular conclusion. More in general, they provide evidence that the source of the process leading to a certain conclusion C is unreliable, defective, or false (Melis, 2016, p. 271–2). Consider a general connection between a premise and a conclusion: usually collective vaccination lets unvaccinated children stay safe. This can be rephrased as a conditional claim: if an unvaccinated child is protected by collective vaccination, then she can stay safe. We might question the validity of this conditional: in principle, everybody should be vaccinated and an unvaccinated child can stay safe as long as vaccination is collective. However, if everyone thinks that their child can be unvaccinated and anyway stay safe, the validity of the inference will be undercut (U): vaccination would be no more collective and unvaccinated children could not stay safe. Paradoxically, as in the case of single “free riders”, they can take advantage of vaccination without being vaccinated, as long as they do not undermine the need for vaccination to be collective.
(b) The second role consists of a *prima facie* reason to directly question the conclusion of an inference. For example, Alice believes that collective vaccination also protects unvaccinated people and concludes that unvaccinated children can stay safe. However, Alice learns about Aldo, a child suffering from a serious pathology, who cannot get vaccinated and cannot stay safe since people around him are not vaccinated. Hence, the actual presence of unvaccinated people in Aldo's community is a fact that counts as a premise to be handled with care in reasoning about vaccination. If unvaccinated people are the majority, the premise can become the basis to directly rebut (R) Alice's conclusion. Hence, an additional premise that is liable to rebut Alice's belief is one that strongly suggests the falsity of the believed proposition.

The different roles of premises in reasoning about vaccination also show that, depending on the proportion of unvaccinated people, our reasoning path can be more or less nuanced. Reasoning on a single unvaccinated child and reasoning on the fact that Aldo, the unvaccinated little boy, is surrounded by unvaccinated people, makes a relevant difference. In the latter case, we can rebut Alice's conclusion that unvaccinated children can stay safe, as the presence of an unvaccinated majority is evidence against it. So, premises that threaten to defeat a conditional are potentially more revisionary in scope, while premises liable to rebut a conclusion may highlight some local problems in our reasoning as we may discover that such a conclusion actually does not follow (Kelly, 2014).

Medical expertise involves navigating across a web of potential invalidating conditions, which create epistemically uncertain scenarios: a case in point is surely diagnostic reasoning (Salis and Ervas, 2021). This equals almost to a proviso that premises are to be handled with special care within these reasoning tasks. Therefore, medical expertise, in the context of uncertain reasoning tasks, consists of the ability to identify the correct connections between safer and more uncertain premises, and to rule out the potential invalidating conditions for the expert's hypotheses (and not only in cases concerning vaccination). Many factors can be helpful in such uncertain scenarios: the usual route is provided by procedures of belief-revision between interlocutors, where their dialogue is an implicit means order to check the mutual appropriateness and consistency of one's beliefs. This is a reliable way to understand how we manage to improve our reasoning skills, but it is not easy, and it is also fallible. However, an interesting alternative option may come from recent studies on metaphor as a reasoning device in argumentative texts.

### Metaphor as a reasoning device

In the case of institutional communication aiming at improving vaccination rates, it is of outstanding importance to let people understand how experts handle reasoning about vaccination in uncertain scenarios. Metaphors might be a good candidate to let people grasp the importance of cooperative action for vaccination and reason about the many ways the individual adherence to the therapy might affect the collective endeavor of vaccination, in the perspective of a shared health responsibility. In this perspective, metaphors need to be carefully selected based on their potentiality to fit (or even enhance) the reasoning required by an argumentative discourse on diagnosis and the consequent need for therapy adherence in the case of vaccine-preventable diseases. Previous literature showed that metaphors are never neutral because they entail a framing effect, “often including specific attitudes and evaluations” (Semino et al., 2018, p. 32), that influence the reasoning process (see Thibodeau et al., 2019 for a review). In the conceptual theory of metaphor (CMT), metaphor shapes how we reason about the world (Lakoff, 2004), first of all, because conceptual mapping is inherently selective: some aspects of the source domain are emphasized while other aspects are downplayed. The emphasized aspects guide the reasoner to a specific argumentative path. In a series of experiments where a metaphorical text on crime was presented, metaphor proved to guide participants' reasoning and how they “gather information to make ‘well-informed' decisions” (Thibodeau and Boroditsky, 2011, p. 10). In the field of health communication, Hauser and Schwarz (2015) showed that the metaphor of “enemy” for cancer, when compared to neutral frames, reduces participants' willingness to limit their behaviors considered correlated to cancer likelihood. In the case of vaccine communication, Scherer et al. (2015) found that metaphors of “beast”, “riot”, “army”, or “weed” to describe flu increased participants' intentions to get vaccinated when compared to “virus” as a literal counterpart.

Within the domain of everyday dilemmas, strong framing effects were reported for the kind of reasoning required by the text (see for instance Tversky and Kahneman, 1983, on a dilemma involving a disease outbreak). Interestingly, in a metaphor-based task for the resolution of dilemmas, metaphors proved to have no (or even undesired) communicative effects, especially when increasing ambiguity or uncertain reasoning (Robins and Mayer, 2000). The specific line of reasoning required the participants to solve the dilemma dominated over possible metaphorical inferences on the target: as the authors concluded, “the metaphor is unnecessary in cases in which a metaphor interferes with the reasoner's understanding process” (Robins and Mayer, 2000, p. 61). Under this respect, far from scaffolding and covertly driving reasoning as claimed by Thibodeau et al. (2017), the reasoner's understanding process would rather drive the eventual effects of the metaphorical framing. Still, it is an open question whether and how the reasoning process and the metaphorical framing might creatively interact. For instance, in simple deductive tasks, Ervas et al. (2018) showed that conventional metaphors, whose meaning is lexicalized and so familiar that goes unnoticed by participants, can be revitalized to hold a plausible conclusion of the argument, while novel metaphors, whose meaning is so new and creative that are fast recognized as metaphorical, rather lead to think about alternative conclusions.

Previous studies tested reasoning about “safe” scenarios where participants were asked to draw a conclusion and checked where their inferences were influenced by the metaphorical description of the issue. Scherer et al. (2015) tested the effects of the metaphorical framing of flu description on vaccine intentions, without considering different uncertain reasoning scenarios. No study on the effects of metaphors for vaccination on reasoning in uncertain scenarios has been conducted, even though it should be required in vaccine communication, especially during pandemic times. Previous literature in corpus studies provided mixed results and questioned the use of metaphors in health communication (Skelton et al., 2002; Macagno and Rossi, 2019), showing that it might be a source of ambiguity and failures in understanding. Thus, the role and usefulness of metaphor in reasoning about vaccination remain an open problem. It is still unclear when and how metaphors might be effective reasoning devices to improve patients' understanding of a specific diagnostic process, for instance in the case of vaccination, as well as adherence to therapy.

## The study

A valid alternative to CMT (but still controversial, see Deignan, 2011; Gibbs, 2011, 2015; Müller, 2011; Charteris-Black, 2012; Beger, 2019 for criticism) to study the effects of metaphor in vaccine communication might come from the deliberate metaphor theory (DMT) (Steen, 2008, 2011, 2017). In this perspective, the deliberate use of a metaphor, i.e., the intentional use of a metaphor *as* a metaphor, would be fundamental to draw attention to a target issue (vaccination) with specific communicative aims (compliance). For instance, a novel deliberate metaphor (especially when *direct*, as for instance when introduced by “like” explicitly prompting a comparison between domains) can be fruitfully employed as a rebuttal analogy in argumentative discourse, while a conventional metaphor would not serve this job (Van Poppel, 2020a).

In previous work (Salis and Ervas, 2021), we suggested that in health communication a metaphor might be intentionally selected for the sake of the argumentative aims, because its properties are helpful in explaining a (collective) health problem, making some reasons for a diagnosis clearer, offering an alternative on a system of commonplaces associated with the illness, and/or soliciting a belief revision in the illness itself. In our view, an argumentatively apt metaphor, i.e., a metaphor whose properties favor the reasoning process that leads to understanding the diagnosis and therapy management, might be crucial. As *patient trust* is related to the success of health communication (Fallowfield and Jenkins, 1999; Thom, 2001), an argumentatively apt metaphor might become fundamental not only for the doctor–patient relationship but also for the confidence in experts and the institution in general. However, to the best of our knowledge, no empirical study on the effects of metaphor use on trust in experts and institutions in the case of vaccine communication in uncertain scenarios has been conducted.

### Experimental hypotheses

We proposed to participants a study to understand whether and when a novel metaphor (“the beehive”, see Biss, 2014; Ervas, 2018), extended *via* the relevant property (“cooperative”) in an argumentative text on vaccination in uncertain scenarios, can be an effective reasoning and communicative device for laypeople's understanding of vaccination as a vital collective endeavor. The study aims at understanding the communicative effects of metaphors in uncertain reasoning situations, in terms of persuasion, emotional impact, trust in experts/institutions, and vaccination intentions. We proposed the following main hypotheses:

H1: A novel metaphor for vaccination, extended *via* a relevant property for the reasoning task, entails stronger communicative effects than its literal counterpart, as it is explicitly intended to make the collective vaccination dynamic more intelligible for laypeople.
H2: Reasoning situations concerning uncertain scenarios enhance the comprehension of the text on vaccination when compared to safe reasoning scenarios, as they make possible defeating conditions (not envisaged in safe scenarios) explicit.

We expected that participants grasped the importance of collective vaccination and health-related behaviors, especially in uncertain scenarios with potential *rebutting defeaters*, which offer a prima facie reason (a fact) to directly question the conclusion.

As extended metaphors were explicitly intended to highlight the target (vaccination), under an *alternative perspective*, they drive the reader's attention toward the conclusion, especially in the case of premises that are more liable to make the reasoning scenario uncertain. This function should not be achieved by their literal counterparts, as they do not help *per se* defeasible reasoning in providing an alternative perspective. We, therefore, hypothesized that the metaphorical frame interacts with the reasoning process type, in the following way:

H3: Extended metaphors are more effective as both communicative and reasoning devices than their literal counterparts in uncertain scenarios, where reasoning is usually defeasible. Especially in the case of possible undercutting defeaters, concerning a potential withdrawal of the *main inference*, the explicit relevant property of the metaphor for defeasible reasoning might make it easier for the interlocutor to discern whether the inference is valid or not. This happens since undercutting defeaters question the validity of a conditional, and conditionals (e.g., if X, then Y) are the best locutions we possess in order to make inferences explicit, and so easily evaluable. Thus, when the metaphor is extended *via* a relevant property to support or even enhance the defeasible reasoning process on vaccination, it can better *achieve some communicative effects* in the argumentative discourse, in terms of comprehension, persuasion, and trust in the experts (and the institutions they represent).

### Experimental design

The empirical study had a 3 × 2 experimental design: 3 “reasoning” conditions (a. safe reasoning scenarios; b. uncertain reasoning scenarios – undercutting-type, U; c. uncertain reasoning scenarios – rebutting-type, R) × 2 “wording frame” conditions [(a) metaphor; (b) literal counterpart].

Six groups of participants were therefore provided with an argumentative text on vaccination in the following six conditions:

1) Texts presenting a safe reasoning scenario with either a metaphor (MS) or its literal counterpart (LS);
2) Texts presenting an uncertain reasoning scenario (undercutting-type) with either a metaphor (MU) or its literal counterpart (LU);
3) Texts presenting an uncertain reasoning scenario (rebutting-type) with either a metaphor (MR) or its literal counterpart (LR).

### Participants

A total of 196 adults (152F, 44M; M_*age*_ = 27.97 years; SD_*age*_ = 10.40) participated in the study. All participants spoke Italian as their first language and signed an informed consent form indicating that they understood the nature of their participation in the study, which was approved by the Ethics Committee of the University of Cagliari (n. 0107679, 05/06/2020). A description of the six groups of participants, each assigned with a single condition, is provided in Table 1.

**Table 1:** Participants' demographic table.

**Condition**	**Literal (L)**			**Metaphorical (M)**		
	**Standard** **(*N =* 32)**	**Undercutting** **(*N =* 31)**	**Rebutting** **(*N =* 33)**	**Standard** **(*N =* 30)**	**Undercutting** **(*N =* 33)**	**Rebutting** **(*N =* 37)**
Gender	24F/8M	24F/7M	24F/9M	22F/8M	27F/6M	31F/6M
Mean age (SD)	32.25 (10.03)	31.64 (10.32)	27.88 (10.14)	26.70 (10.92)	22.97 (5.72)	26.76 (11.86)

Most participants declared to gather information about vaccination by consulting a medical competent expert (43.1%) or *via* institutional websites (31.8%); a minority *via* non-institutional websites and social networks (3.7%), informal discourses with relatives or friends (5.5%), scientific journals (6.2%). Some participants declared to be not informed at all about vaccination (6.9%).

### Materials

Previous literature showed that especially when the metaphor is put at the *beginning of a text*, it encourages drawing inferences consistent with the frame provided by the metaphor itself. The source domain knowledge from which to draw the inferences (Thibodeau et al., 2017) is activated even more when the metaphor is *extended* along the text (Keefer et al., 2014; Thibodeau, 2016), *via* a relevant property (“collective” in the literal conditions *vs*. “collaborative” in the metaphorical conditions) for reasoning about potentially defeating scenarios. The material was a set of six argumentative texts on vaccination (one per condition, see Appendix for the material in Italian). Table 2 presents the texts translated into English, with the potential defeaters in italics and the extended metaphor in bold (as well as its literal counterpart in the literal conditions).

**Table 2:** Argumentative texts on vaccination (translation into English).

	**Safe reasoning (S)**	**Uncertain reasoning- Undercutting-type (U)**	**Uncertain reasoning- Rebutting-type (R)**
**Literal (L)**	The expert talked about collective immunity concerning viral epidemics and told that the vaccination of everyone is a fundamental requirement against contagion. In particular, the doctor insisted on defining the collective effort for everyone's vaccination as that of the **components** of a **group**, where the collective counts more than the individual. The speech concerned the idea of **collective** commitment of all citizens. The expert concluded emphasizing how much we are dependent on each other in the context of sanitary emergencies.	The expert talked about collective immunity concerning viral epidemics and told that the vaccination of everyone is a fundamental requirement against contagion. In particular, the doctor insisted on defining the collective effort for everyone's vaccination as that of the **components** of a **group**, where the collective counts more than the individual. The speech concerned the idea of **collective** commitment of all citizens. *The expert stressed that even an unvaccinated child can stay safe if people around him are vaccinated*. The expert concluded emphasizing how much we are dependent on each other in the context of sanitary emergencies.	The expert talked about collective immunity concerning viral epidemics and told that the vaccination of everyone is a fundamental requirement against contagion. In particular, the doctor insisted on defining the collective effort for everyone's vaccination as that of the **components** of a **group**, where the collective counts more than the individual. The speech concerned the idea of **collective** commitment of all citizens. *The expert provided the example of Aldo, a child suffering from a serious pathology, who cannot get vaccinated and cannot stay safe since people around him are not vaccinated*. The expert concluded emphasizing how much we are dependent on each other in the context of sanitary emergencies.
**Metaphorical (M)**	The expert talked about collective immunity concerning viral epidemics and told that the vaccination of everyone is a fundamental requirement against contagion. In particular, the doctor insisted on defining the collective effort for everyone's vaccination as that of the **bees in a beehive**, where the collective counts more than the individual. The speech concerned the idea of **collaborative** commitment of all citizens. The expert concluded emphasizing how much we are dependent on each other in the context of health emergencies.	The expert talked about collective immunity concerning viral epidemics and told that the vaccination of everyone is a fundamental requirement against contagion. In particular, the doctor insisted on defining the collective effort for everyone's vaccination as that of the **bees in a beehive**, where the collective counts more than the individual. The speech concerned the idea of **collaborative** commitment of all citizens. *The expert stressed that even an unvaccinated child can stay safe if people around him are vaccinated*. The expert concluded emphasizing how much we are dependent on each other in the context of health emergencies.	The expert talked about collective immunity concerning viral epidemics and told that the vaccination of everyone is a fundamental requirement against contagion. In particular, the doctor insisted on defining the collective effort for everyone's vaccination as that of the **bees in a beehive**, where the collective counts more than the individual. The speech concerned the idea of **collaborative** commitment of all citizens. *The expert provided the example of Aldo, a child suffering from a serious pathology, who cannot get vaccinated and cannot stay safe since people around him are not vaccinated*. The expert concluded emphasizing how much we are dependent on each other in the context of health emergencies.

### Procedure

The data were collected at the beginning of the COVID-19 pandemic before vaccines were made available to the population. The data were collected *via* six online forms, one for each condition. The participants signed the informed consent and information about gender, age, language, and education was collected. The participants were then asked to read the instructions and fill out a questionnaire, with answers on a 1 to 7 Likert Scale, ranging from 1 as “not at all” to 7 as “very much”.

In the first part of the questionnaire, all the forms presented the conclusion of the argumentative texts as a stand-alone sentence (the same for all conditions): “In the context of health emergencies we are dependent on each other”. The participants were asked to rate how much they agree with the statement. Then, on a separate screen, the questionnaire presented the same sentence as the conclusion of the argumentative text (different for each condition, see Table 2), asking the participants how much it was logically acceptable when considering the premises of the text.

In the second part of the questionnaire, all the forms presented the relative argumentative text followed by a series of questions, each one on a separate screen. The questions were focused on text comprehension as well as on its perceived communicative effects (see Table 3 for Measures) at *individual* and *collective* levels, as the evaluation of the argumentative texts on vaccination could involve reasoning not only on possible personal vaccination behavior but also its link with the possible collective vaccination behavior. First of all, we aimed to measure whether metaphor actually made the texts easier to understand, though introducing a meaning ambiguity (the literal meaning vs. the metaphorical meaning of the source). We then selected the measures, and related questions, from previous literature on the metaphorical framing effects on vaccination (Scherer et al., 2015) in terms of persuasion, perceived control, and vaccination intentions. In light of the possible effects of extended metaphor on source credibility and trust (Bowers and Osborn, 1966; Reinsch, 1974; Baake, 2003; Brugman et al., 2019), we added specific measures for participants' trust in the expert's advice, on experts and the institutions they represent, we deemed to be highly relevant for vaccine communication.

**Table 3:** Description of measures and related response categories.

**Response categories**	**Description**
Agreement	Participants indicated their level of agreement with the statement (= the conclusion as a single sentence).
Logical acceptability	Participants indicated whether the conclusion was logically acceptable, given the premises of the text.
Understandability	Participants indicated how much the text was easy to understand (at the individual and collective level).
Ambiguity	Participants indicated whether they found meaning ambiguities in the text.
Emotional impact	Participants indicated how much they found the advice presented in the text emotionally appealing (at the individual and collective level).
Convincingness	Participants indicated how much they found convincing (at the individual and collective level) the advice presented in the text.
Safety	Participants indicated the feeling of safety (at the individual and collective level) in a health situation like that described in the text.
Control	Participants indicated the feeling of control (at the individual and the collective level) in the health situation described in the text.
Commitment	Participants indicated the level of commitment to vaccination (at the individual and collective level).
Trust in Experts	Participants indicated the level of trust in experts (at the individual and collective level).
Uptake of the expert's' advice	Participants indicated the (individual and collective) level of uptake of the expert's advice.
Trust in Institutions	Participants indicated the level of trust in institutions.
Vaccination Intentions	Participants indicated the likelihood that they would get vaccinated in the upcoming winter season.

The final section of the questionnaire gathered information about participants' previous general vaccination behavior and beliefs about (flu) vaccination, the impact of COVID-19, and an eventual COVID-19 vaccination on their lives (see Appendix for the questionnaire).

## Results

All the data collected are available at the following OSF address. A 2 (literal vs. metaphorical) × 3 (standard *vs*. undercutting *vs*. rebutting reasoning) ANOVA was performed for all dependent variables (response categories in Table 3). Effect sizes were estimated using partial eta squared () for ANOVA (Gravetter and Wallnau, 2006). Power analysis was conducted in G^*^Power 3.1.9.4 (Faul et al., 2007) prior to the study. The G^*^Power results showed that a sample size of 196 would produce a power value of 0.80, considering an alpha of 0.05, the effect size of 0.260 (η^2^ partial of 0.05). Table 4 presents the statistical results of all main effects for the metaphorical frame, reasoning structure, and interactions for each response category.

**Table 4:** Effects of the metaphorical frame, the reasoning structure, and their interaction on response categories.

	**Framing**			**Reasoning structure**			**Metaphorical frame: Reasoning structure**		
**Response categories**	***F*-value**	***p-*value**	** $\eta_{p}^{2}$ **	***F*-value**	***p-*value**	** $\eta_{p}^{2}$ **	***F*-value**	***p-*value**	** $\eta_{p}^{2}$ **
Agreement	0.003	0.956	0.000	1.967	0.143	0.020	0.715	0.490	0.007
Logical Acceptability	3.070	0.081	0.016	2.685	0.071	0.027	0.678	0.509	0.007
Understandability_I	8.421	0.004	0.042	4.094	0.018	0.041	0.833	0.437	0.009
Understandability_C	12.006	0.001	0.059	3.337	0.038	0.034	0.149	0.861	0.002
Ambiguity	1.572	0.212	0.008	7.418	0.001	0.072	0.390	0.678	0.004
Emotional impact_I	2.486	0.117	0.013	4.706	0.010	0.047	1.034	0.358	0.011
Emotional impact _C	4.482	0.036	0.023	6.604	0.002	0.065	1.264	0.285	0.013
Convincingness_I	14.297	0.000	0.070	3.802	0.024	0.038	2.354	0.098	0.024
Convincingness _C	8.310	0.004	0.042	6.639	0.002	0.065	0.067	0.936	0.001
Safety_I	11.539	0.001	0.057	0.657	0.520	0.007	3.286	0.040	0.033
Safety_C	5.192	0.024	0.027	4.025	0.019	0.041	0.649	0.524	0.007
Control_I	4.206	0.042	0.022	0.450	0.638	0.005	0.498	0.608	0.005
Control_C	4.822	0.029	0.025	2.286	0.104	0.023	2.188	0.115	0.023
Commitment_I	0.065	0.799	0.000	2.540	0.082	0.026	2.566	0.079	0.026
Commitment _C	0.354	0.553	0.002	4.722	0.010	0.047	1.024	0.361	0.011
Trust in Experts_I	2.257	0.135	0.012	0.935	0.394	0.010	3.392	0.036	0.034
Trust in Experts_C	7.989	0.005	0.040	1.436	0.240	0.015	1.525	0.220	0.016
Uptake of Experts' Adv._I	3.088	0.080	0.016	1.770	0.173	0.018	4.268	0.015	0.043
Uptake of Experts' Adv _C	4.468	0.036	0.023	3.923	0.021	0.040	2.698	0.070	0.028
Trust in Institutions _I	0.198	0.657	0.001	0.365	0.695	0.004	2.338	0.099	0.024
Trust in Institutions_C	0.381	0.538	0.002	1.839	0.162	0.019	3.448	0.034	0.035
Vaccination Intentions_I	3.309	0.070	0.017	2.706	0.069	0.028	0.244	0.784	0.003
Vaccination Intentions_C	1.415	0.236	0.007	2.835	0.061	0.029	0.384	0.682	0.004

The metaphorical framing effect was especially significant for the overall text understandability (both at an individual and at a collective level), the emotional impact (even though only at a collective level), the convincingness of the text (both at an individual and at a collective level), the perceived safety (both at an individual, and at a collective level), the feeling of control over the health situation (both at an individual and at a collective level), the collective trust in expertise and uptake of experts' advice. No significant effect of the metaphorical frame was found on participants' responses for the other categories.

The effect of the reasoning structure was instead found especially for participants' perceived ambiguity of meaning, but also for the overall text understandability (both at an individual, and at a collective level), the convincingness of the text (both at an individual, and at a collective level), the collective perceived safety, commitment to vaccination and uptake of experts' advice. No significant effect of the reasoning structure was found for the other response categories.

The metaphorical framing × reasoning structure interaction was significant for the personal feeling of safety, individual trust in expertise, uptake of experts' advice, and collective trust in institutions. Means and SDs for each response category, for the metaphorical frame, and for reasoning structure conditions are shown in Table 5. The results of *post hoc* comparisons using the Tukey HSD test are also reported in Table 5. The results revealed a significant difference among the reasoning conditions for the understandability at the individual level and the collective perceived safety and uptake of the experts' advice. In particular, the undercutting reasoning condition significantly differs from the other conditions for the understandability and commitment at a collective level, perceived ambiguity, the emotional impact at an individual level, and convincingness at both an individual and a collective level. A significant difference was observed between the rebutting and the undercutting condition for the emotional impact at a collective level.

**Table 5:** Means and SDs of response categories scores by framing and reasoning structure condition.

	**Framing**		**Reasoning structure**		
	**Literal**	**Metaphorical**	**Standard**	**Undercutting**	**Rebutting**
	**Mean (SD)**	**Mean (SD)**	**Mean (SD)**	**Mean (SD)**	**Mean (SD)**
Agreement	6.00 (1.30)	6.01 (1.23)	5.74 (1.56) ^a^	6.11 (1.11) ^a^	6.14 (1.05) ^a^
Logical Acceptability	5.77 (1.48)	6.13 (1.38)	5.74 (1.63) ^a^	5.81 (1.50) ^a^	6.27 (1.14) ^a^
Understandability_I	6.11 (1.26)	6.58 (0.93)	6.02 (1.49) ^**a**^	6.55 (0.80) ^**b**^	6.47 (0.94) ^**ab**^
Understandability_C	4.30 (1.38)	4.97 (1.27)	4.27 (1.46) ^a^	4.77 (1.27) ^ab^	4.86 (1.30) ^b^
Ambiguity	2.63 (1.81)	2.28 (1.80)	3.05 (2.08) ^a^	2.52 (1.75) ^ab^	1.86 (1.40) ^b^
Emotional impact_I	3.75 (2.05)	4.23 (1.87)	3.47 (2.12) ^a^	3.94 (1.80) ^ab^	4.51 (1.88) ^b^
Emotional impact _C	4.08 (1.68)	4.57 (1.37)	3.98 (1.53) ^a^	4.09 (1.56) ^a^	4.86 (1.43) ^b^
Convincingness_I	4.47 (1.72)	5.30 (1.40)	4.50 (1.66) ^a^	4.88 (1.70) ^ab^	5.26 (1.42) ^b^
Convincingness _C	4.40 (1.48)	4.94 (1.09)	4.21 (1.33) ^a^	4.73 (1.36) ^ab^	5.03 (1.15) ^b^
Safety_I	3.69 (1.71)	4.52 (1.80)	3.92 (1.72) ^a^	4.31 (2.01) ^a^	4.10 (1.67) ^a^
Safety_C	3.84 (1.41)	4.30 (1.34)	3.69 (1.34) ^a^	4.39 (1.56) ^b^	4.13 (1.20) ^ab^
Control_I	4.17 (1.94)	4.69 (1.59)	4.37 (1.94) ^a^	4.61 (1.87) ^a^	4.33 (1.57) ^a^
Control_C	4.52 (1.59)	5.02 (1.58)	4.42 (1.77) ^a^	4.86 (1.49) ^a^	5.01 (1.50) ^a^
Commitment_I	5.93 (1.63)	5.98 (1.78)	5.56 (1.85) ^a^	6.22 (1.54) ^a^	6.06 (1.68) ^a^
Commitment _C	6.31 (1.46)	6.43 (1.12)	5.98 (1.69) ^a^	6.44 (1.25) ^ab^	6.66 (0.76) ^b^
Trust in Experts_I	5.18 (1.89)	5.53 (1.61)	5.13 (1.85) ^a^	5.38 (1.91) ^a^	5.54 (1.51) ^a^
Trust in Experts_C	4.34 (1.28)	4.80 (0.92)	4.37 (1.15) ^a^	4.66 (1.29) ^a^	4.69 (0.94) ^a^
Uptake of Experts' Adv._I	5.46 (1.85)	5.87 (1.63)	5.35 (1.88) ^a^	5.70 (1.81) ^a^	5.91 (1.55) ^a^
Uptake of Experts' Adv _C	4.08 (1.29)	4.42 (0.96)	3.94 (1.20) ^a^	4.48 (1.28) ^b^	4.33 (0.86) ^ab^
Trust in Institutions _I	5.32 (1.65)	5.41 (1.52)	5.23 (1.50) ^a^	5.45 (1.81) ^a^	5.41 (1.44) ^a^
Trust in Institutions_C	3.85 (1.38)	3.97 (1.23)	3.81 (1.34) ^a^	4.17 (1.43) ^a^	3.77 (1.12) ^a^
Vaccination Intentions_I	4.34 (2.25)	4.91 (2.00)	4.11 (2.38) ^a^	4.78 (2.04) ^a^	4.96 (1.93) ^a^
Vaccination Intentions_C	4.86 (1.82)	5.18 (1.77)	4.58 (1.92) ^a^	5.16 (1.77) ^a^	5.30 (1.65) ^a^

Means followed by the same letter at the same row are not significantly different, p < 0.05, according to the pairwise t-test with Bonferroni correction.

Means and SDs for all response category scores, for standard, undercutting, and rebutting reasoning conditions, separately for literal and metaphorical frames, are shown in Table 6. Follow-up univariate analyses, further examining reasoning differences were run separately for literal and metaphorical frames.

**Table 6:** Mean (M) and standard deviation (SD) for each condition and response category.

	**Literal**			**Metaphorical**		
	**Standard**	**Undercutting**	**Rebutting**	**Standard**	**Undercutting**	**Rebutting**
**Response category**	**Mean (SD)**	**Mean (SD)**	**Mean (SD)**	**Mean (SD)**	**Mean (SD)**	**Mean (SD)**
Agreement	5.63 (1.62)^a^	6.26 (1.21) ^a^	6.12 (0.93) ^a^	5.87 (1.50) ^a^	5.97 (1.02) ^a^	6.16 (1.17) ^a^
Logical acceptability	5.44 (1.68) ^a^	5.61 (1.63) ^a^	6.24 (0.97) ^a^	6.07 (1.53) ^a^	6.00 (1.37) ^a^	6.30 (1.29) ^a^
Understandability_I	5.69 (1.64) ^a^	6.45 (0.81) ^b^	6.21 (1.08) ^ab^	6.37 (1.25) ^a^	6.64 (0.78) ^a^	6.70 (0.74) ^a^
Understandability_C	4.00 (1.55) ^a^	4.35 (1.28) ^a^	4.55 (1.28) ^a^	4.57 (1.33) ^a^	5.15 (1.15) ^a^	5.14 (1.27) ^a^
Ambiguity	3.34 (1.86) ^a^	2.55 (1.71) ^ab^	2.00 (1.64) ^b^	2.73 (2.29) ^a^	2.48 (1.80) ^a^	1.73 (1.15) ^a^
Emotional impact_I	3.53 (2.27) ^a^	3.58 (1.88) ^a^	4.12 (2.00) ^a^	3.40 (1.99) ^a^	4.27 (1.68) ^ab^	4.86 (1.72) ^b^
Emotional impact _C	4.00 (1.80) ^a^	3.74 (1.73) ^a^	4.48 (1.48) ^a^	3.97 (1.22) ^a^	4.42 (1.32) ^a^	5.19 (1.31)^b^
Convincingness_I	4.03(1.77) ^a^	4.19 (1.82) ^ab^	5.15 (1.37) ^b^	5.00 (1.39) ^a^	5.52 (1.33) ^a^	5.35 (1.48) ^a^
Convincingness _C	3.97 (1.51) ^a^	4.42 (1.52) ^a^	4.79 (1.32) ^a^	4.47 (1.07) ^a^	5.03 (1.13) ^ab^	5.24 (0.95) ^b^
Safety_I	3.56 (1.76) ^a^	3.45 (1.96) ^a^	4.03 (1.38) ^a^	4.30 (1.62) ^a^	5.12 (1.73) ^a^	4.16 (1.91) ^a^
Safety_C	3.56 (1.27) ^a^	4.00 (1.69) ^a^	3.97 (1.24) ^a^	3.83 (1.42) ^a^	4.76 (1.35) ^b^	4.27 (1.17) ^ab^
Control_I	4.25 (2.14) ^a^	4.16 (2.13) ^a^	4.09 (1.59) ^a^	4.50 (1.72) ^a^	5.03 (1.51) ^a^	4.54 (1.54) ^a^
Control_C	3.88 (1.66) ^a^	4.87 (1.48) ^b^	4.82 (1.47) ^b^	5.00 (1.72) ^a^	4.85 (1.52) ^a^	5.19 (1.52) ^a^
Commitment_I	5.38 (1.79) ^a^	5.97 (1.80) ^ab^	6.42 (1.09) ^b^	5.77 (1.92) ^a^	6.45 (1.23) ^a^	5.73 (2.02) ^a^
Commitment _C	5.88 (1.84) ^a^	6.26 (1.59) ^ab^	6.79 (0.48) ^b^	6.10 (1.54) ^a^	6.61 (0.79) ^a^	6.54 (0.93) ^a^
Trust in Experts_I	4.69 (1.87) ^a^	5.00 (2.39) ^ab^	5.82 (1.07) ^b^	5.60 (1.73) ^a^	5.73 (1.26) ^a^	5.30 (1.79) ^a^
Trust in Experts_C	4.28 (1.33) ^a^	4.23 (1.48) ^a^	4.52 (1.03) ^a^	4.47 (0.94) ^a^	5.06 (0.93) ^b^	4.84 (0.83) ^ab^
Uptake of Experts' Adv._I	4.91 (2.02) ^a^	5.23 (2.17) ^ab^	6.21 (0.93) ^b^	5.83 (1.62) ^a^	6.15 (1.25) ^a^	5.65 (1.92) ^a^
Uptake of Experts' Adv _C	3.81 (1.38) ^a^	4.06 (1.41) ^a^	4.36 (1.03) ^a^	4.07 (0.98) ^a^	4.88 (1.02) ^b^	4.30 (0.70) ^a^
Trust in Institutions _I	5.13 (1.60) ^a^	5.13 (2.17) ^a^	5.70 (0.98) ^a^	5.33 (1.40) ^a^	5.76 (1.35) ^a^	5.16 (1.72) ^a^
Trust in Institutions_C	4.00 (1.41) ^a^	3.77 (1.61) ^a^	3.79 (1.11) ^a^	3.60 (1.25) ^a^	4.55 (1.15) ^b^	3.76 (1.14) ^a^
Vaccination Intentions_I	3.75 (2.48) ^a^	4.45 (2.34) ^a^	4.82 (1.84) ^a^	4.50 (2.26) ^a^	5.09 (1.70) ^a^	5.08 (2.02) ^a^
Vaccination Intentions_C	4.34 (1.94) ^a^	4.94 (2.00) ^a^	5.30 (1.40) ^a^	4.83 (1.90) ^a^	5.36 (1.54) ^a^	5.30 (1.87) ^a^

Within the same condition (literal vs. metaphorical), means followed by the same letter at the same row are not significantly different, p < 0.05, according to the pairwise t test with Bonferroni correction.

In the literal condition, the results revealed a significant difference among the reasoning conditions for understandability at an individual level. In particular, the undercutting reasoning condition significantly differs from the other conditions for the perceived ambiguity, the convincingness at an individual level, the commitment at both an individual and collective level, trust in experts, and the uptake of the experts' advice at an individual level.

In the metaphorical condition, the results revealed a significant difference among the reasoning conditions for the collective perceived safety and trust in experts. In particular, the undercutting reasoning condition significantly differs from both the other conditions for the emotional impact at an individual level and the convincingness at a collective level. A significant difference between the undercutting and the rebutting reasoning conditions was found for the collective emotional impact and between the undercutting and the standard reasoning conditions for the collective uptake of the experts' advice and trust in institutions.

To further investigate the effect of the metaphorical framing, independent two sample *t*-tests were run separately for standard, undercutting, and rebutting reasoning conditions. In the case of the standard reasoning condition, the results revealed a significant difference between the literal and the metaphorical conditions for the response categories of convincingness at an individual level (*t* = −2.387; *p* = 0.020), and control at a collective level (*t* = −2.618; *p* = 0.011). In the case of undercutting reasoning condition, a significant difference between the literal and the metaphorical conditions was found for the response categories of understandability at a collective level (*t* = −2.624; *p* = 0.011), convincingness at an individual level (*t* = −3,341; *p* = 0.001), safety at an individual level (*t* = −3.617; *p* = 0.001), trust in experts at a collective level (*t* = −2.721; *p* = 0.008), uptake of the experts' advice at both an individual (*t* = −2.105; *p* = 0.039) and a collective level (*t* = −2.653; *p* = 0.010), trust in institutions at a collective level (*t* = −2.220; *p* = 0.030). In the case of rebutting reasoning condition, a significant difference between the literal and the metaphorical conditions was found for the response categories of understandability at an individual level (*t* = −2.233; *p* = 0.029), and emotional impact at a collective level (*t* = −2.112; *p* = 0.038).

## Discussion

Metaphor proves to be very useful to let laypeople understand a complex health issue in the case of vaccination: when compared to literal descriptions of vaccination, the beehive metaphor makes the message easier to understand. The results confirm previous literature as to what concerns the general communicative potential of metaphor (Sopory and Dillard, 2002; Ottati and Renstrom, 2010): independently of the reasoning structure of the text, participants thought that the metaphorical description of vaccination could be more persuasive not only for themselves but also for the majority of people, especially for what concerns the emotional impact. Metaphors can indeed have many functions in both interpersonal and mass communication and be mediated by multiple psychological mechanisms. First of all, metaphor activates information directly connected to the communication topic, as it attributes some relevant properties of the source to the target. In the process, it can favor the understanding of the communication topic, but also influence people's attitudes and emotive evaluation toward the communication topic (Thibodeau, 2016; Ervas et al., 2021). In the case of vaccine communication, participants' attitude and evaluations were indeed influenced by the metaphorical description of vaccination as a collaborative endeavor, especially for what concerned their perceived feeling of safety and control over the health situation at both a personal and collective level. The first main hypothesis (H1) that extended metaphors, explicitly intended to promote the vaccination uptake *via* a relevant property (“collaborative”) for the argumentative text, elicit stronger communicative effects than their literal counterpart, is thereby confirmed.

The results also confirmed the second main hypothesis (H2) that uncertain reasoning scenarios are relevant for the comprehension of the text on vaccination. Not only the overall understandability of the text was higher in uncertain reasoning scenarios when compared to safe reasoning scenarios, but also its convincingness at both an individual and collective level. Texts based on an uncertain reasoning structure make explicit possible defeaters which are instead not envisaged in safe scenarios. Making possible defeating conditions explicit, texts based on uncertain reasoning structure allow a better grasp of how vaccination works, resulting in even clearer than texts based on a safe reasoning structure. Besides anticipating possible defeaters, the texts based on uncertain reasoning scenarios propose a solution in the conclusion at the collective level, thus enhancing also the collective feeling of perceived safety and the possible collective commitment and adherence to vaccination.

The persuasive effect of uncertain reasoning scenarios is significantly stronger in the case of rebutting defeaters, perhaps due to the stronger emotional impact at a collective level (however, the reference to Aldo as suffering from a pathology may give rise to a ‘victim effect' —introducing another emotional factor— and this may be a limitation of this study). Texts based on uncertain scenarios *via* rebutting defeaters are perceived not only as more understandable and leading to vaccination commitment at a personal level, but also as more convincing and emotionally appealing than the safe reasoning situations at a collective level. These texts were indeed based on the single story of the child, Aldo, “suffering from a serious pathology, who cannot get vaccinated and cannot stay safe since people around him are not vaccinated”. Drawing on a concrete and potentially “more dangerous” case when compared to both the other cases, the text may be experienced also as more emotionally appealing and the communicative effect of the text may be enhanced by a reasoning process that also exploits aspects of imaginative self-identification and/or empathy to promote vaccination (Shelby and Ernst, 2013). Especially when compared to texts based on safe reasoning scenarios, the rebutting case is indeed highly visual (without having the contraindications of a real picture, see Nyhan et al., 2014) and easier to emotionally share (Dubé et al., 2020). Previous literature also shows indeed that presenting factual descriptions are more successful communication strategy for vaccination commitment than presenting undercutting literal arguments against anti-vaccination myths (Horne et al., 2015; Greenberg et al., 2017). On the contrary, texts based on uncertain situations *via* undercutting defeaters are thought to increase the feeling of safety and the uptake of experts' advice at a collective level when compared to texts based on safe reasoning conditions. Differently from the reasoning conditions with rebutting defeaters, those with an undercutting defeater instead present a general condition that could undermine the conclusion, thus having stronger communicative effects.

However, neither a main metaphorical framing effect nor a main defeasible reasoning effect was found on participants' acceptance of the conclusion, trust in institutions, and vaccination intentions (see Table 4). The metaphorical frame does not directly influence reasoning (as instead was found by Thibodeau and Boroditsky, 2011, 2013), but provides a more nuanced set of communicative effects. While metaphor was found to influence participants' acceptance of the conclusion in the case of syllogisms in narrow contexts (Ervas et al., 2018), this effect seems lost in the case of wider contexts, where the persuasive effects of the text prevail over the argument evaluation. Furthermore, metaphor use is not a predictor of higher vaccination intentions, as instead was found by Scherer et al. (2015). Presumably, just reading a text presenting a metaphor for vaccination is unlikely to directly produce a change in vaccination intentions, which are strongly linked to participants' previous beliefs on vaccination. As already discussed, the vaccination commitment and adherence depend on a more nuanced set of reasoning conditions, being different at a personal and collective level. The results also disconfirm the main interaction between the metaphorical framing and the reasoning structure on the acceptability of the text conclusion and the overall set of communicative effects. However, even though the main effects of both the metaphorical frame and the defeasible reasoning on the acceptability of the conclusion are overall independent, the results partially confirm H3, showing a significant interaction effect in the case of the individual feeling of safety, trust in expertise and uptake of experts' advice, especially in the case of reasoning with undercutting defeaters, and collective trust in institutions, so important during the pandemic.

Metaphor descriptions of vaccination and defeasible reasoning support each other especially in the undercutting reasoning condition, as the metaphor was extended in the text *via* the relevant property (“collaborative”) underpinning a more general connection to the conclusion. The undercutting defeater would be the more relevant premise to invert the direction of the main inference to the conclusion, which is instead granted by both the conclusion of the text and the extended metaphor. Metaphors can indeed “influence the degree to which the recipient generates pro- or counter-arguments” (Ottati and Renstrom, 2010, p. 787) and, especially when extended, metaphor can support the *inference* and the *direction* of elaboration of the argument where it appears. In this case, the metaphor —*via* its extended property— directs attention toward the most relevant feature from an argumentative point of view and supports the reasoning process, by activating an expectancy of the conclusion and making the overall text coherent. In directing attention to the most relevant property for the sake of argument, the metaphor can also have a corresponding directional effect on attitudes *toward the topic* of communication. Indeed, from a communicative perspective, this interaction between extended metaphor and reasoning with undercutting defeaters is effective in making the interlocutor feel reassured by the expert and trust her expertise, possibly connected to the increased uptake of experts' advice. The case of reasoning with rebutting defeaters is somewhat different in this respect, as it is based on a concrete case where the metaphorical relevant property is itself undermined (by the anti-vaccination collective behavior).

Previous studies supported the idea that metaphor can also influence people's attitudes and evaluation toward the communication source, thus affecting the credibility of the speaker and favoring attitude change (Bowers and Osborn, 1966). The communicator credibility view suggests that communicators who use metaphors are judged more credible than ones who use literal language (Reuchamps et al., 2018; Brugman et al., 2019). The reason is that novel metaphors may point out unexpected analogies, thus possibly leading to creative thinking and/or revision of (previous) untenable beliefs. As the success of health communication is intrinsically linked with patient trust (Fallowfield and Jenkins, 1999; Thom, 2001), metaphor use might turn out to be essential not only to the proper comprehension of vaccination but also to therapy adherence (Rossi and Macagno, 2020). However, previous studies provided mixed results on metaphor's enhancement of the source credibility (Reinsch, 1974; Sopory and Dillard, 2002) and our study supports the idea that “well-used” metaphors in specific reasoning scenarios rather than metaphors *tout court* facilitate persuasion. As argued above, it is not the ability to highlight relevant similarities between the beehive and vaccination *per se* that automatically makes the extended metaphor a good reasoning strategy for the overall argument. In the cases of vaccination analyzed in this study, both communicative and reasoning competencies seem to be required to boost trust and vaccination commitment (Thom, 2001), which finally is at the core of the vaccine hesitancy problem (Larson et al., 2018; Sondagar et al., 2020).

Previous studies on the deliberate use of scientific metaphors in academic discourse (Beger, 2019) showed that there is an asymmetry in expertise between the expert and the addressee which might entail a mismatch in the uptake of the metaphor. Of course, the listener's knowledge of the target domain is relevant, as it influences the path of possible sound inferences on the target issue. The mismatch might be simply due to the constitutive expert/laypeople asymmetry in terms of domain knowledge, which might be overcome by appropriate communicative/argumentative strategies (Bigi, 2016). However, pre-existing knowledge about the target (Reuchamps et al., 2018) or strong ideological views about the target (Landau et al., 2014) could make the metaphor unlikely to change how people reason about the target domain, as well as their attitudes or behavior (Ottati and Renstrom, 2010). For instance, in the case of vaccination, Scherer et al. (2015, p. 37) concluded that the effects of the metaphorical frames “were found among individuals who occasionally receive flu vaccinations but not among individuals who never or always receive flu vaccinations”. Further research should be done on the influence of previous beliefs on individual differences in vaccination behavior: a trustful relationship with experts and institutions needs to be achieved even more so to counter vaccine hesitancy (Yaquib et al., 2014), which is not confined to people refusing (or encouraging others to refuse) vaccination.

## Conclusion

The results of the study provided an overall picture of the communicative and reasoning competencies far more nuanced than expected in using metaphor to promote vaccination. The study should be extended as it is limited to a geographical area, and also presented in another time-framework, when not (directly) influenced by the COVID-19 outbreak. Further research should also consider conventional and non-extended metaphors as well as emotive metaphors (i.e., metaphors having positive/negative valence) to compare the metaphorical communicative effects, especially in terms of emotional impact and persuasion, on vaccination uptake. Also, alternative reasoning scenarios should be considered, to further understand the different roles of rebutting vs. undercutting defeaters in reasoning about vaccination.

The metaphor does not automatically increase vaccination commitment and trust. Not even defeasible reasoning, as a specific competence of experts', can do that. Rather, both communicative and defeasible reasoning competences are needed to enhance trust in immunization, with possible different outcomes at an individual and collective level. As in other fields of science communication in general and health communication in particular, metaphor can be an opportunity for a good argument, constructing social bonding but also committing to both the risks and responsibilities of communication (Frezza, 2016). Depending on the argumentative structure and the kind of defeasible envisaged alternatives, metaphor can also present an imagined scenario (with its possible features and consequences) we might or not adhere to (Ervas, 2019), influencing our uptake of the overall argument.

## Data availability statement

The dataset analyzed for this study can be found at the following OSF address: https://osf.io/9hy8z/?view_only=34d4c8d38cf74bff966205f04f35032f.

## Ethics statement

The studies involving human participants were reviewed and approved by the Ethics Committee of the University of Cagliari (no. 0107679, 05/06/2020). The patients/participants provided their written informed consent to participate in this study.

## Author contributions

FE and RF: experimental design. FE and PS: construction of the materials and manuscript writing. FE, PS, and RF: data collection. CS: data analysis. All authors provided feedback on the draft and approved the final version of the manuscript.

## Funding

FE gratefully acknowledges the support of the University of Cagliari (Premialità 2019, no. 246/19C, 28.11.2019) and Fondazione Banco di Sardegna (research grant no. F72F20000420007).

## Conflict of interest

The authors declare that the research was conducted in the absence of any commercial or financial relationships that could be construed as a potential conflict of interest.

## Publisher's note

All claims expressed in this article are solely those of the authors and do not necessarily represent those of their affiliated organizations, or those of the publisher, the editors and the reviewers. Any product that may be evaluated in this article, or claim that may be made by its manufacturer, is not guaranteed or endorsed by the publisher.
