# Supplementary material for: Exploring metaphor's communicative effects in reasoning on vaccination
Source: Front Psychol. 2022 Nov 18;13:1027733. doi: 10.3389/fpsyg.2022.1027733 (PMC9716206; doi:10.3389/fpsyg.2022.1027733)
Supplement: Supplementary file 1 [file Data_Sheet_1.docx]

**Appendix**

|  | **Standard (S)** | **Undercutting (U)** | **Rebutting** **(R)** |
| --- | --- | --- | --- |
| **Literal (L)** | L’esperto ha parlato di immunità collettiva riguardo alle epidemie virali e ha affermato che la vaccinazione di tutti è un requisito fondamentale contro il contagio. In particolare, l’esperto ha insistito nel definire lo sforzo collettivo per la vaccinazione come quello dei **componenti di un gruppo**, dove il collettivo conta più del singolo. Il suo discorso si è incentrato sull’aspetto dell’impegno **collettivo** di tutti i cittadini. L’esperto ha concluso mettendo in evidenza quanto siamo dipendenti gli uni dagli altri nel contesto delle emergenze sanitarie. | L’esperto ha parlato di immunità collettiva riguardo alle epidemie virali e ha affermato che la vaccinazione di tutti è un requisito fondamentale contro il contagio. In particolare, l’esperto ha insistito nel definire lo sforzo collettivo per la vaccinazione come quello dei **componenti di un gruppo**, dove il collettivo conta più del singolo. Il suo discorso si è incentrato sull’aspetto dell’impegno **collettivo** di tutti i cittadini. *L’esperto ha ricordato che anche un bambino non vaccinato può stare al sicuro se le persone intorno a lui sono vaccinate.* L’esperto ha concluso mettendo in evidenza quanto siamo dipendenti gli uni dagli altri nel contesto delle emergenze sanitarie. | L’esperto ha parlato di immunità collettiva riguardo alle epidemie virali e ha affermato che la vaccinazione di tutti è un requisito fondamentale contro il contagio. In particolare, l’esperto ha insistito nel definire lo sforzo collettivo per la vaccinazione come quello dei **componenti di un gruppo**, dove il collettivo conta più del singolo. Il suo discorso si è incentrato sull’aspetto dell’impegno **collettivo** di tutti i cittadini. *L’esperto ha fatto l’esempio di Aldo, un bambino con una grave patologia, che non può essere vaccinato e non può stare al sicuro perché le persone intorno a lui non sono vaccinate.* L’esperto ha concluso mettendo in evidenza quanto siamo dipendenti gli uni dagli altri nel contesto delle emergenze sanitarie. |
| **Metaphorical (M)** | L’esperto ha parlato di immunità collettiva riguardo alle epidemie virali e ha affermato che la vaccinazione di tutti è un requisito fondamentale contro il contagio. In particolare, l’esperto ha insistito nel definire lo sforzo collettivo per la vaccinazione come quello delle **api di un alveare**, dove il collettivo conta più del singolo. Il suo discorso si è incentrato sull’aspetto dell’impegno **collaborativo** di tutti i cittadini. L’esperto ha concluso mettendo in evidenza quanto siamo dipendenti gli uni dagli altri nel contesto delle emergenze sanitarie. | L’esperto ha parlato di immunità collettiva riguardo alle epidemie virali e ha affermato che la vaccinazione di tutti è un requisito fondamentale contro il contagio. In particolare, l’esperto ha insistito nel definire lo sforzo collettivo per la vaccinazione come quello delle **api di un alveare**, dove il collettivo conta più del singolo. Il suo discorso si è incentrato sull’aspetto dell’impegno **collaborativo** di tutti i cittadini. *L’esperto ha ricordato che anche un bambino non vaccinato può stare al sicuro se le persone intorno a lui sono vaccinate.* L’esperto ha concluso mettendo in evidenza quanto siamo dipendenti gli uni dagli altri nel contesto delle emergenze sanitarie. | L’esperto ha parlato di immunità collettiva riguardo alle epidemie virali e ha affermato che la vaccinazione di tutti è un requisito fondamentale contro il contagio. In particolare, l’esperto ha insistito nel definire lo sforzo collettivo per la vaccinazione come quello delle **api di un alveare**, dove il collettivo conta più del singolo. Il suo discorso si è incentrato sull’aspetto dell’impegno **collaborativo** di tutti i cittadini. *L’esperto ha fatto l’esempio di Aldo, un bambino con una grave patologia, che non può essere vaccinato e non può stare al sicuro perché le persone intorno a lui non sono vaccinate*. L’esperto ha concluso mettendo in evidenza quanto siamo dipendenti gli uni dagli altri nel contesto delle emergenze sanitarie. |

Table A1. Argumentative texts on vaccination in Italian

| **Response categories** | **Questions (Italian)** | **Questions (English)** |
| --- | --- | --- |
| Agreement | Quanto sei d'accordo? | How much do you agree? |
| Logical acceptability | Viste le premesse, pensi che la conclusione del testo sia logicamente accettabile? | Given the premises, do you think the conclusion of the text is logically acceptable? |
| Understandability | Quanto è comprensibile questo testo per te/per la maggioranza delle persone? | How much is this text understandable for you/the majority of people? |
| Ambiguity | Secondo te il testo presenta qualche ambiguità di significato? | In your opinion, does the text present meaning ambiguities? |
| Emotional impact | Quanto ti sembra che il testo faccia leva sulle tue emozioni/sulle emozioni della maggioranza delle persone? | How much do you believe that the text is emotionally appealing for you/for the majority of people? |
| Convincingness | Quanto ti sembra che il testo risulti convincente/per la maggioranza delle persone? | How much do you believe that the text is convincing/for the majority of people? |
| Safety | Quanto ti ha rassicurato il messaggio trasmesso dall'esperto?/Quanto pensi che si possa sentire rassicurata la maggioranza delle persone dal messaggio trasmesso dall'esperto? | How much the message of the expert made you feel safe?/How much do you think that the message of the expert can make the majority of people feel safe? |
| Control | Quanta possibilità di controllo pensi di poter avere a livello personale su una situazione sanitaria come quella descritta nel testo?/Quanto pensi che una situazione sanitaria come quella descritta dal testo possa essere controllata a livello collettivo? | How much do you think that you can be in control in a health situation like that described in the text?/ How much do you think a health situation like that described in the text can be controlled at the collective level? |
| Commitment | In una situazione sanitaria come quella descritta nel testo, quanto pensi che incida il tuo impegno personale a vaccinarti/l'impegno di tutti a vaccinarsi? | How much do you think your personal/everyone’s commitment to vaccination counts in a health situation like that described in the text? |
| Trust in Experts | Quanta fiducia hai nei suggerimenti dati dall'esperto nel testo?/Quanta fiducia potrebbe avere la maggioranza delle persone nei suggerimenti dati dall'esperto nel testo? | How much do you trust the expert’s advice in the text?/ How much could the majority of people trust the expert’s advice in the text? |
| Uptake of the expert’s’ advice | Quanto ritieni di essere pronto ad accettare i consigli che l'esperto ha dato nel testo?/Quanto ritieni che la maggioranza delle persone siano pronte ad accettare i consigli che l'esperto ha dato nel testo? | How much do you believe that you are ready to accept the expert’s advice in the text?/How much do you believe that people are ready to accept the expert’s advice in the text? |
| Trust in Institutions | Quanto ritieni di avere fiducia nelle prescrizioni istituzionali, qui rappresentate dall'esperto?Quanto ritieni che la maggioranza delle persone abbia fiducia nelle prescrizioni istituzionali, qui rappresentate dall'esperto? | How much do you trust institutional prescriptions, here represented by the expert?/How much do you believe that the majority of people trust institutional prescriptions, here represented by the expert? |
| Vaccination Intentions | Quanto ritieni di dover fare il vaccino contro l'influenza stagionale la prossima stagione invernale?/Quanto ritieni che la maggior parte delle persone debba fare il vaccino contro l'influenza stagionale la prossima stagione invernale? | How much do you believe that you should get vaccinated against flu for the next winter season?/How much do you think that the majority of people should get vaccinated against flu for the next winter season? |
| **Questions (Italian)** | | **Questions (English)** |
| Behaviour toward vaccination | Hai fatto qualche vaccino negli ultimi tre anni? | Did you get any vaccine in the last three years? |
| Agreement on vaccination | Quanto sei d'accordo con la pratica della vaccinazione in generale? | How much do you agree in general with the practice of vaccination? |
| Opinion on great pharmaceutical companies | Secondo te, quanto influenti sono le grandi case farmaceutiche sulle politiche di vaccinazione? | In your opinion, how much are great pharmaceutical companies influential on vaccination politics? |
| Behaviour toward flu vaccination | Ti sei vaccinato per l'influenza negli ultimi tre anni? | Did you get vaccine against flu in the last three years? |
| Impact of an eventual COVID-19 vaccine | In che misura pensi che un vaccino contro il Covid-19 cambierebbe la tua vita/la vita di tutti? | How much do you think that a vaccine against Covid-19 would change your/everyone’s life? |
| COVID-19 Impact on opinion on vaccination | Quanto ha cambiato le tue opinioni sulla vaccinazione la situazione di emergenza legata al Covid-19? | How much the health emergency related to Covid-19 has changed your opinion about vaccination? |

Table A2. Questionnaire in Italian and translation into English
